# Patterns of steroid use in patients with established rheumatoid arthritis commencing treatment with biologic or targeted synthetic DMARDs

**DOI:** 10.1093/rap/rkaf130

**Published:** 2025-12-22

**Authors:** Michael Stadler, Shae Bindra, Annie Cheung, Chuan Fu Yap, James Bluett, Darren Plant, Nisha Nair, Kimme Hyrich, Ann Morgan, Anthony G Wilson, John D Isaacs, Anne Barton

**Affiliations:** Centre for Genetics and Genomics Versus Arthritis, University of Manchester, Manchester, UK; NIHR Manchester Biomedical Research Centre, Manchester University Foundation Trust, Manchester, UK; Centre for Genetics and Genomics Versus Arthritis, University of Manchester, Manchester, UK; NIHR Manchester Biomedical Research Centre, Manchester University Foundation Trust, Manchester, UK; Centre for Genetics and Genomics Versus Arthritis, University of Manchester, Manchester, UK; Centre for Genetics and Genomics Versus Arthritis, University of Manchester, Manchester, UK; NIHR Manchester Biomedical Research Centre, Manchester University Foundation Trust, Manchester, UK; Centre for Genetics and Genomics Versus Arthritis, University of Manchester, Manchester, UK; NIHR Manchester Biomedical Research Centre, Manchester University Foundation Trust, Manchester, UK; Centre for Genetics and Genomics Versus Arthritis, University of Manchester, Manchester, UK; NIHR Manchester Biomedical Research Centre, Manchester University Foundation Trust, Manchester, UK; Centre for Epidemiology Versus Arthritis, University of Manchester, Manchester, UK; School of Medicine, University of Leeds, Leeds, UK; NIHR Leeds Biomedical Research Centre, Leeds Teaching Hospitals NHS Trust, Leeds, UK; UCD School of Medicine and Medical Science, Conway Institute, University College, Dublin, Ireland; Translational and Clinical Research Institute, Newcastle University, Newcastle upon Tyne, UK; NIHR Newcastle Biomedical Research Centre, Newcastle upon Tyne Hospitals NHS Foundation Trust, Newcastle upon Tyne, UK; Centre for Genetics and Genomics Versus Arthritis, University of Manchester, Manchester, UK; NIHR Manchester Biomedical Research Centre, Manchester University Foundation Trust, Manchester, UK

**Keywords:** rheumatoid arthritis, biologics, glucocorticoids

## Abstract

**Objectives:**

To investigate the clinical use of steroids in established RA.

**Methods:**

A cohort of RA patients commencing treatment with biologic or targeted synthetic (b/ts) DMARDs was followed prospectively, with clinical data recorded pre-baseline and at the 3, 6 and 12-month follow-up. Patients were included in this analysis if they had completed the first year of follow-up and had data on steroid use available for at least one follow-up. The proportions of patients receiving steroids at different timepoints were compared and further differentiated between continued (receiving steroids at two consecutive timepoints) and newly started treatment. Lastly, mixed linear effect models were used to assess the relationship between clinical factors and steroid use.

**Results:**

The cohort (*N* = 1846) had a median disease duration of 7 years and, across each timepoint, ≈30% of patients received steroids, up to two-thirds of which continued treatment from a preceding follow-up. At 12 months, the proportion of patients continuing treatment decreased, but more patients started steroid treatment (*P* < 0.001). Linear mixed effects modelling further showed that steroid use was more common in patients who had required pre-baseline steroids [odds ratio (OR) 1.44 (95% CI 1.38, 1.49)] and in those on later-stage treatments [OR 1.15 (95% CI 1.08, 1.23)].

**Conclusion:**

Despite the introduction of b/tsDMARDs, steroid use in this cohort continued over the first year of follow-up, particularly in patients with more severe RA. Together with previous data, this further highlights the need for future research and trials to better understand the right course of steroid administration to maximize efficacy and limit adverse side effects.

Key messages

- Patients in this cohort continued to be prescribed steroids despite the introduction of targeted therapies.
- More severe disease was associated with increased odds of being prescribed steroids.
- Additional research is required to better understand the right dose and mode of steroid administration.

## Introduction

Synthetic glucocorticoids (GCs), first introduced in the 1940s and also simply known as steroids, are an integral component of clinical treatment strategies for RA [1]. RA is characterized by synovial joint inflammation and steroids provide fast-acting systemic anti-inflammatory effects to alleviate symptoms [2]. GCs used in RA include but are not limited to prednisolone, dexamethasone and methylprednisolone, which are administered either orally, intravenously or via joint/muscle injection [3]. While steroids are highly effective in managing RA symptoms [2], long-term use is associated with a range of adverse effects, including a significant increase in cardiovascular risk [4, 5], an already common comorbidity and a leading cause of death among patients with RA [6, 7].

By comparison, DMARDs are considered to have a safer side-effect profile [8]. DMARDs reduce symptoms and slow disease progression and are favoured over steroids [1]. However, DMARDs, specifically conventional DMARDs (cDMARDs), can take up to 6 months to become effective [3], and early treatment is vital to maintain a patient’s overall quality of life and to prevent irreparable joint damage, which may otherwise lead to functional impairment and disability [9, 10]. With the most recent update to the National Institute for Health and Care Excellence (NICE) guidance in 2018 [11], it was recommended to consider prescribing steroids when a patient with early RA is starting a new cDMARD, to bridge this gap to treatment onset. Notably, this UK recommendation, and current European recommendation on bridging [12], are specifically for cDMARDs. Biologic and targeted synthetic DMARDs (b/tsDMARDs) have comparably faster average onset than cDMARDs [1], but evidence for the use of steroids as a bridging therapy during b/tsDMARD initiation is lacking, leading to an absence of guidelines for this clinical scenario [13].

Beyond the addition of bridging in early RA, steroid treatment remains more generally recommended to manage disease flares [11], but at a low dose and for the shortest clinically feasible time [14]. Despite this, a number of studies over the past 2 decades have found that steroid use remains common, often even after multiple months of DMARD treatment [1, 15–19]. While much of the existing evidence is focused on early RA [20], in the UK, b/tsDMARDs are typically reserved for patients with moderate or severe RA who have had an inadequate response to intensive treatments with cDMARDs already [11], at which point TNF inhibitors (TNFis) are generally tried first before other targeted therapies are considered [21]. To better understand the role of steroids in the clinical care of this patient group, data from the Biologics in Rheumatoid Arthritis Genetics and Genomics Study Syndicate (BRAGGSS) [22] were used to investigate patterns of how steroid use changes over the course of 1 year from b/tsDMARD initiation in relation with different treatments and patients’ disease activity. Additionally, differences between patients who started treatment before and after the latest NICE guidance update were explored to gauge the impact of these changes on later disease stages.

## Methods

### Dataset

To investigate the use of steroids in clinical practice, data were obtained from BRAGGSS, a prospective observational cohort of consenting patients with a clinical diagnosis of RA commencing treatment with a b/tsDMARD under a consultant rheumatologist in the UK [22]. Demographics and clinical information were collected at an initial assessment appointment (baseline) and at 3, 6 and 12 months after treatment initiation. Recent steroid use before b/tsDMARD initiation (pre-baseline) was defined based on information provided by the treating clinician, which included details on steroid route, dose and duration/frequency. Additionally, the BRAGGSS study collected information from the treating clinician on whether patients received oral steroids (yes/no) and whether patients received parenteral (intravenously/injected) steroids (yes/no) as part of their ongoing treatment at each follow-up appointment, but further details on the specific steroid and dose were not collected. BRAGGSS commenced in 2008 based on existing registry data from the British Society of Rheumatology’s Biologics Register [23], received ethical approval from the Northwest 6 Central Manchester South Research Ethics Committee (COREC 04/Q1403/37) and all patients provided written informed consent.

### Statistical analysis

Patients were included in this analysis if they had completed 12 months of follow-up and had data on steroid use available for at least one follow-up timepoint. The cohort was characterized based on demographics and baseline clinical information. The last major update to the NICE treatment guidance was published in 2018 [11], and to investigate whether this had an impact on steroid use in the clinic, patients were further stratified based on treatment start date, with one group starting treatment before 2018 and the other after. Demographics, baseline clinical characteristics and proportions of steroid use between the two groups were compared via independent sample *t*-tests, Mann–Whitney U tests or the χ^2^ statistic, as appropriate. The proportions of patients using steroids at each follow-up were calculated and compared between oral and parenteral (intravenously or via joint/muscle injection) steroids. Steroid use was further differentiated between patients who were continuing treatment (also received steroids at the preceding timepoint) and those who were starting new treatment (did not receive steroids at the preceding timepoint). Differences in steroid use and mode of steroid use between the first follow-up (3 months) and the last follow-up (12 months) were assessed using the McNemar test [24].

Following NICE guidance [11], disease activity was measured using the four-component DAS, calculated for 28 joints and using CRP levels as a marker for active inflammation (DAS28_CRP_) [25]. To assess the impact of steroids on disease activity beyond their initial fast-acting effects, paired *t*-tests were first used to see whether disease activity remained decreased in patients who received steroids by comparing their DAS28_CRP_ before (3/6 months) and after their steroid course (6/12 months). Then independent sample *t*-tests were used to compare the DAS28_CRP_ after treatment (6/12 months) between patients who received steroids and those who did not to see whether steroids improved disease activity on par with patients who did not require steroids.

Mixed linear effects models [26] were used to longitudinally assess steroid use (yes/no) over the course of the follow-up period. A random effect was used to account for repeated measures of the same individual and a fixed effect was used to account for differences in timepoint. To account for differences in treatment, the model was adjusted for type of b/tsDMARD the patient was receiving (TNFi or non-TNFi) using a fixed effect and an interaction term with the follow-up. This baseline model was further adjusted using fixed effects for the following baseline clinical factors: whether the patient was starting their first course of b/tsDMARDs, whether the patient had received pre-baseline steroids, whether the patient started treatment before 2018, whether the patient was RF positive and whether the patient received concomitant cDMARDs (methotrexate, hydroxychloroquine or sulfasalazine) when they started treatment. Significant effects were retained in the model, which was then further adjusted based on disease activity. Forward selection based on the Akaike information criterion (AIC) [27], using the DAS28_CRP_ and its components as well as the physician’s global health assessment (PGA) as candidate predictors, was used to iteratively add individual predictors to the model until the model’s AIC no longer improved significantly (ΔAIC <10). Disease activity factors vary over time and were therefore also modelled through a fixed effect plus an interaction with the follow-up. The effects of different factors on steroid use were assessed based on the *P*-value and the odds ratio (OR).

Lastly, patients who received steroids despite being in clinical remission (DAS28_CRP_ <2.4 [28]) were identified. To investigate why patients may have been kept on steroids despite being in remission, linear mixed models were used to look for differences across disease activity factors between patients in remission who received further steroids and those who did not.

Statistical analysis was carried out in python (version 3.5.1). Independent sample *t*-tests, paired *t*-tests, Mann–Whitney U tests and χ^2^ tests were carried out using the ttest_ind, ttest_rel, mannwhitneyu and chi2_contingency functions from the scipy package (version 1.6.2). McNemar tests were carried out using the mcnemar function from the statsmodels package (version 0.13.2). Mixed linear effect models were fitted using the mixedlm function from the statsmodels package (version 0.13.2).

## Results

A total of 1846 patients from BRAGGSS had completed the entire 1-year follow-up period and had data on steroid use for least one follow-up. The demographics and baseline characteristics for the study cohort are shown in Table 1. The cohort had an average age of ≈57 years and was predominantly female (≈76%), with a median disease duration of ≈7 years and an average baseline DAS28_CRP_ of 5.7. There were 1263 patients (≈68%) on TNFi treatment and 583 patients (≈32%) on later-stage, non-TNFi treatments (Supplementary Table S1).

**Table 1.** Baseline demographics and clinical characteristics overall and stratified by year of treatment start.

Characteristics	Full cohort (N = 1846)	<2018 (n = 1673)	≥2018 (n = 173)	P-value	Missing, %
Age, years, mean (s.d.)	57.2 (12)	57.3 (12)	56.7 (11.8)	0.5	0.4
Female, n (%)	1410 (76.4)	1267 (75.7)	143 (82.7)	0.05	–
BMI, median (IQR)	27.5 (23.4–31.6)	27.6 (23.4–31.8)	27.2 (23.3–31.1)	0.4	22.3
Disease duration, years, median (IQR)	7.2 (6.1)	7.2 (6.2)	6.5 (5.3)	0.07	2.3
Age RA onset, years, median (IQR)	48 (38.5–57.5)	48 (38.5–57.55)	48 (40–56)	0.6	1.8
RF positive, n (%)	1078 (69.5)	988 (70.3)	90 (62.1)	**0.049**	16
First b/tsDMARD, n (%)	1317 (72.1)	1187 (71.8)	130 (75.6)	0.3	1.1
Group TNFi, n (%)	1263 (68.4)	1153 (68.9)	110 (63.6)	0.2	–
Concomitant cDMARD, n (%)	1549 (84.8)	1412 (84.6)	137 (87.3)	0.4	1.1
Pre-baseline steroids, n (%)	625 (33.9)	279 (32.8)	36 (47.4)	**0.004**	–
DAS28_CRP_, mean (s.d.)	5.7 (4.9–6.5)	5.7 (4.8–6.6)	5.5 (4.8–6.2)	**0.002**	9.1
CRP (mg/l), median (IQR)	10.4 (−0.8–21.6)	10.4 (−1–21.8)	10.4 (0.9–19.9)	0.6	4.3
SJC28, median (IQR)	7 (4–10)	7.5 (4.5–10.5)	7 (4.5–9.5)	0.2	4
TJC28, median (IQR)	14 (8.5–19.5)	14 (8.5–19.5)	12 (7.6–16.4)	**0.0003**	4
PtGA (mm), median (IQR)	76 (64.6–87.4)	75 (63.5–86.5)	80 (70.1–89.9)	0.4	5.2
PGA (mm), median (IQR)	70 (57.5–82.5)	70 (57–83)	75 (65–85)	**0.001**	25.7

Group TNFi: proportion of patients who started treatment with TNF inhibitors; concomitant cDMARD: proportion of patients who received concomitant cDMARD treatment at baseline; SJC28: 28-joint swollen joint count; TJC28: 28-joint tender joint count; PtGA: patient’s global health assessment; PGA: physician’s global health assessment.

Age and DAS28_CRP_ were compared using independent sample *t*-tests; other continuous factors were compared using Mann–Whitney U tests; categorical data are presented as the percentage of non-missing data and were compared using the χ^2^ statistic.

*P*-value for the statistical test comparing values between the year strata; bold type indicates significance at *P* < 0.05.

A total of 33.9% of patients had received steroid treatment pre-baseline; 17.1% received oral steroids, 15.6% received parenteral steroids and 0.5% received both. The median oral dose was 7.5 mg [interquartile range (IQR) 5–10; 30.8% missing] and the median parenteral dose was 80 mg (IQR 23.1–136.9; 17.2% missing). Pre-baseline steroid use was more frequent in the non-TNFi group (*P* < 0.001), and excluding samples with missing DAS28_CRP_, there was no significant difference in baseline DAS28_CRP_ between patients who had recently received steroids [mean 5.72 (s.d. 0.87) and those who did not [mean 5.68 (s.d. 0.84); *P* = 0.3; *n* = 1678]. The earliest treatment in this study’s cohort commenced in 2001 and the latest commenced in 2020. There were 173 patients (9%) who started treatment in or after 2018 and, compared with patients who started treatment before 2018, they had lower disease activity (*P* = 0.002) and were more likely to have received pre-baseline steroids (*P* = 0.002), but there was no significant difference in treatment groups (*P* = 0.2) or other treatment factors (Table 1). Additionally, there was no significant difference in the mode of steroid administration between the two groups (Supplementary Table S2).

Across all timepoints, the proportion of steroid use was roughly 30%, except patients who started treatment since 2018 received fewer steroids at 6 months than the rest of the cohort (Table 2). The overall proportion of steroid use between the first and last follow-up increased by 3.5%, which was statistically significant (McNemar *P* = 0.046). At the same time, the share of oral steroids decreased from 45.5 to 35.5%, but the share of parenteral steroids increased from 31.7 to 38.8%, and the proportion of patients who received both oral and parenteral steroids increased from 6.9 to 11.1% (Supplementary Table S3). This difference in mode of steroid use between the first and last follow-up was statistically significant (*P* < 0.001).

**Table 2.** Steroid use across the follow-up period, overall and stratified by year of treatment start.

Month	N	Cohort	Steroids, n (%)	P_O_	Continued, n (%)	Started, n (%)	P_C_	Missing, %
3	1787	Full	521 (29.2)		339 (65.1)	182 (34.9)		0
	1616	<2018	476 (29.5)	0.4	310 (65.1)	166 (34.9)	1	0
	171	≥2018	45 (26.3)		29 (64.4)	16 (35.6)		0
6	1757	Full	527 (30)		329 (64.9)	178 (35.1%)		3.8
	1589	<2018	**489 (30.8)**	**0.04**	304 (64.8)	165 (35.2)	1	4.1
	168	≥2018	38 (22.6)		25 (65.8)	13 (34.2)		0
12	1714	Full	560 (32.7)		320 (59.1)	221 (40.9)		3.4
	1557	<2018	512 (32.9)	0.6	299 (60.4)	196 (39.6)	0.07	3.3
	157	≥2018	48 (30.6)		21 (45.7)	25 (54.3)		4.2

*N*: number of patients with data on steroid use; Continued: patients who received steroids who also received steroids at the preceding timepoint; Started: patients who received steroids who did not receive steroids at the preceding timepoint; *P*_O_: *P*-value for the χ^2^ statistic comparing the overall proportion of steroid use between the year strata, *P*_C_: *P*-value for the χ^2^ statistic comparing the proportion of continued *vs* newly started steroid treatment between the year strata; Missing: percentage of patients on steroids with missing data on steroid use at the preceding timepoint.

Bold typeface indicates significance at *P* < 0.05.

Proportion of steroid use is presented relative to non-missing data.

**Figure 1.**
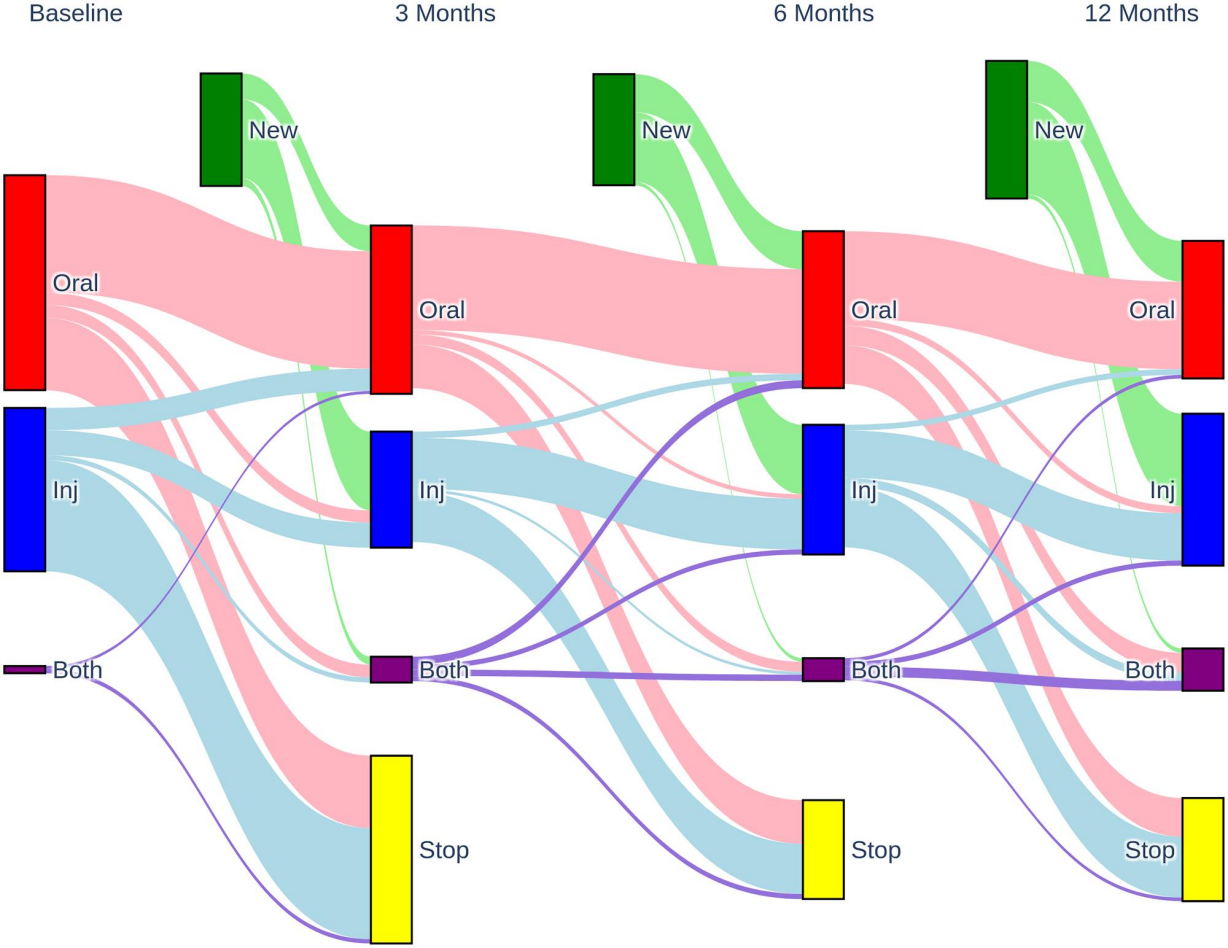
Visualization of the change in steroid regimen over the course of the follow-up period

Approximately two-thirds of patients who received steroid treatment continued from a previous dose, and there were no differences between patients who started treatment since 2018 and the rest of the cohort (Table 2). Although the proportion of patients who continued steroid treatment decreased from 65.1% at 3 months to 59.1% at 12 months, the proportion of patients starting steroid treatment increased from 34.9% at 3 months to 40.9% at 12 months (*P* < 0.001). Overall, oral steroid use was more likely (χ^2^  *P* < 0.001) to persist at subsequent timepoints than parenteral steroids (Fig. 1).

Patients who received steroids at the 3-month follow-up (mean DAS28_CRP_ 3.85 (s.d. 1.33)], had a significant decrease in DAS28_CRP_ at 6 months [3.66 (s.d. 1.42); paired *t*-test *P* = 0.005; *n* = 448]. In contrast, patients who received steroids at 6 months [3.83 (s.d. 1.44)] showed no significant change in their DAS28_CRP_ at 12 months [3.77 (s.d. 1.46); *P* = 0.6; *n* = 445]. Additionally, patients who received steroids at 3 months remained in higher average disease activity at 6 months [3.66 (s.d. 1.42)] than patients who required no steroids [3.27 (s.d. 1.31); independent *t*-test *P* < 0.001; *n* = 1324]. Similarly, patients who received steroids at 6 months remained in higher average disease activity at 12 months [3.77 (s.d. 1.46)] than patients who required no steroids [3.15 (s.d. 1.35); *P* < 0.001; *n* = 1224].

Linear mixed effects models were used to longitudinally investigate steroid use over the course of 1 year from commencing b/tsDMARD treatment. In the baseline model, before adjustments, steroid use was more common at later follow-ups, and patients receiving non-TNFi options had significantly higher odds of steroid use than patients receiving TNFi treatment (Supplementary Table S4). Further adjusting for time-invariant baseline factors showed that pre-baseline steroids increased the odds of steroid use during follow-up, while patients on their first course of b/tsDMARD treatment had reduced odds of using steroids (Supplementary Table S5). Notably there was no association between steroid use and concomitant treatment with cDMARDs and there were no significant differences between choices of cDMARD. All tested disease activity factors were further associated with higher odds of steroid use (*P* < 0.05), and using forward selection based on the AIC, PGA and the DAS28_CRP_ were found to provide additional information for the model. After these adjustments, patients receiving non-TNFi options remained more likely to be using steroids [OR 1.15 (95% CI 1.08, 1.23); *P* < 0.001], but this effect was diminished at 6 and 12 months (Table 3). A higher PGA, as well as higher DAS28_CRP_ at 6 and 12 months, also increased the odds of steroid use, but pre-baseline steroid use remained the strongest predictor of steroid use during follow-up [OR 1.44 (95% CI 1.38, 1.49); *P* < 0.001]. The AIC of this model was high (2766), indicating that much of the variance in steroid use remains unexplained.

**Table 3.** Generalized linear effect model of steroid use across the follow-up period, adjusted for relevant clinical factors.

Variables	β (± SE)	OR (95% CI)	P-value
Intercept	0.045 (**±** 0.046)	1.05 (0.96, 1.14)	0.33
FU_2_	−0.063 (**±** 0.045)	0.94 (0.86, 1.03)	0.16
FU_3_	−0.080 (**±** 0.046)	0.92 (0.84, 1.01)	0.08
nTNFi	**0.142 (±** **0.033)**	**1.15 (1.08, 1.23)**	**<0.001**
nTNFi * FU_2_	**−0.084 (±** **0.035)**	**0.92 (0.86, 0.99)**	**0.018**
nTNFi * FU_3_	**0.098 (±** **0.037)**	**0.91 (0.84, 0.97)**	**0.007**
First b/tsDMARD	−0.020 (**±** 0.028)	0.98 (0.93, 1.04)	0.48
Pre-baseline steroids	**0.363 (±** **0.020)**	**1.44 (1.38, 1.49)**	**<0.001**
PGA	**0.002 (±** **0.001)**	**1.0018 (1.00004, 1.0032)**	**0.01**
PGA * FU_2_	**0.002 (±** **0.001)**	**0.9976 (0.9957, 0.9996)**	**0.018**
PGA * FU_3_	−0.001 (**±** 0.001)	1.00 (1.00, 1.00)	0.17
DAS28_CRP_	0.010 (**±** 0.013)	1.01 (0.98, 1.04)	0.45
DAS28_CRP_ * FU_2_	**0.051 (±** **0.018)**	**1.05 (1.02, 1.09)**	**0.003**
DAS28_CRP_ * FU_3_	**0.060 (±** **0.018)**	**1.06 (1.02, 1.10)**	**<0.001**

β: the parameter estimate and associated standard error (SE); FU_2,3_: the second (6 months) and third (12 months) follow-up, with the first (3 months) serving as the reference category; nTNFi: whether the patient received treatment with TNF inhibitors (no) or other non-TNFi treatment (yes); First b/tsDMARD: whether this was the patient’s first course of b/tsDMARD treatment (yes/no); Pre-baseline steroids: whether the patient had received pre-baseline steroids (yes/no).

*P*-value from a two-tailed *t*-test; bold type indicates significance at *P* < 0.05.

Roughly 16% of patients who received steroids at 3 months were in DAS28_CRP_ remission, which further increased to ≈19% at subsequent timepoints, with no significant difference between patients who started treatment since 2018 and the rest of the cohort (Table 4). Most of these patients received oral steroids, but the share of more intense treatment options increased slightly over the course of the follow-up period (Supplementary Table S6). Using linear mixed effects models, no significant differences in tender joint count (*P* = 0.15), swollen joint count (*P* = 0.81), CRP (*P* = 0.23), patient global assessment (*P* = 0.4) or PGA (*P *= 0.23) were found between patients in remission who received steroids and those who did not.

**Table 4.** Number of patients who received steroids despite being in DAS28_CRP_ remission (DAS28_CRP_ <2.4), overall and stratified by year of treatment start.

Follow-up	Cohort	N	In remission, n (%)	P-value	Missing, %
3 months	Full	398	62 (15.6)	–	23.6
	<2018	368	56 (15.2)	0.7	22.7
	≥2018	30	6 (20)		33.3
6 months	Full	374	71 (19)	–	29
	<2018	349	66 (18.9)	1	28.6
	≥2018	25	5 (20)		34.2
12 months	Full	388	73 (18.8)	–	30.7
	<2018	352	64 (18.2)	0.4	31.2
	≥2018	36	9 (25)		25

N: number of patients who received steroids with non-missing DAS28_CRP_.

Percent missing are relative to the number of patients on steroids.

*P*-value for the χ^2^ statistic comparing remission rates among steroid users between the year strata.

## Discussion

Despite advances in therapy, synthetic GCs, or steroids, have remained an integral part of clinical care for RA [1]. To better understand the current clinical use of steroids in patients with moderate or severe RA, a cohort of patients from the BRAGGSS [22] was used to investigate patterns of steroid use in patients over the course of 1 year from starting treatment with a b/tsDMARD.

It has been suggested that the introduction of DMARDs may improve cardiovascular risk in RA, not just by reducing overall inflammation, but also potentially through a reduction in steroid use [29]. In this cohort, however, the introduction of b/tsDMARDs did not mean that steroid treatment could be stopped; the proportion of overall steroid use remained relatively constant but the intensity of treatment increased over the follow-up period. Similar to early RA [20], we saw that approximately two-thirds of patients who received steroid treatment continued steroids at follow-up. While overall use of oral steroids decreased, the rate of parenteral and dual steroid treatment increased. Additionally, steroid use was more frequent in patients on their second biologic or on higher targeted therapy. This is perhaps not surprising, as the BRAGGSS cohort represents the moderate to severe end of the RA spectrum, with patients in the UK only becoming eligible for b/tsDMARD treatment after inadequate response to intensive treatment with cDMARDs [11]. Updated guidance from 2018 recommends steroids for early RA patients starting a new cDMARD, and, in this cohort, patients who started treatment after the change were more likely to have recently received steroids before b/tsDMARD initiation. However, after starting treatment with b/tsDMARDs, there was no association between concomitant cDMARD treatment and steroid use. Additionally, although patients who started treatment from 2018 had decreased odds of using steroids at the 6-month follow-up, there were no other significant differences in steroid use between the two groups to corroborate this effect. Overall, this suggests that the introduction of bridging in early RA has, so far, had only a minor impact on steroid use at later disease stages, highlighting the need for guidance on steroid use in patients initiating treatment with b/tsDMARDs.

Despite more general guidance recommending the tapering of steroids over time [14], continued steroid use was common, particularly for oral steroids. Longitudinal linear effects models showed that despite their treatment with b/tsDMARDs, patients in this cohort continued to be prescribed steroids. Steroid use has previously been associated with RA disease severity [15], which is further corroborated by this model: patients receiving non-TNFi options, typically reserved for cases where TNFi options have failed as well [21], and those who had recently required steroids before starting their new treatment, had significantly higher odds of using steroids over the follow-up period. Additionally, an increase in disease activity at later timepoints was associated with increased odds of being prescribed steroids, highlighting the clinical role of steroids beyond the initial bridging to DMARD onset. Disease activity improved overall in patients who received steroids but remained higher on average than in patients who did not require steroids. Notably though, RF positivity, associated with harder-to-treat disease [30], was not associated with higher odds of steroid use in this cohort. However, the model explained little of the overall variance in steroid use, which may be explained in part by other factors of disease severity and response to treatment, such as, for example, genetic or environmental factors [31].

Up to one-fifth of patients receiving predominantly oral steroid treatment were found to be in DAS28_CRP_ remission. This highlights an important clinical subgroup of patients with potential opportunities to reduce or stop steroid treatment. No significant differences in disease activity (including the PGA) were found that might have helped explain why some patients in remission received steroids and others did not. This suggests that these patients may have received steroids due to other factors, e.g. because of comorbidities or extra-articular manifestations [32], but this could not be further assessed with the data available in the BRAGGSS.

These findings are consistent with previous studies across different cohorts and treatment stages [1, 15–19] and further corroborate the need for additional research to understand the best dose and route of administration to maximize therapeutic effects while minimizing adverse events and cumulative steroid dosage. Similarly, another recent study of patients initiating b/tsDMARDs found that treatment with very-low-dose steroids (<5 mg/day) frequently continues, even after 3 years on b/tsDMARDs, which was associated with a significant increase in cardiovascular risk but no significant improvement with respect to radiographic progression [33]. From the real-world data included in the current study, patients prescribed oral steroids were more likely to persist with steroids over the follow-up period compared with those receiving parenteral steroids, highlighting a potential missed opportunity to review oral steroid requirements during follow-up. One reason may be that oral steroids can be prescribed by primary care physicians and thus may be overlooked by rheumatologists when patients are reviewed in the clinic, but further research is required to explore the reasons for persistent oral steroid use.

Strengths of this study include the size of the BRAGGSS cohort, including >1800 patients with both moderate and severe RA and a variety of treatments, including newer DMARD agents such as Janus kinase inhibitors. A major limitation, however, is that investigating steroid use was not the main purpose of the BRAGGSS and details on steroid dose at follow-up appointments were not collected. Low-dose steroids (<7.5 mg/day) are commonly used in patients with consistent disease activity [34] and may account for some of the patients on persistent steroid treatment. Regardless, a sizable number of patients receiving steroids had reached remission and would be expected to cease steroid treatment based on current guidance [11]. It is possible that patients with persistent steroid use were being prescribed the treatment for a comorbidity, such as asthma, but we were unable to assess this with the data available. Overall, the clinical rationales for the initiation and maintenance of steroid treatment in this study cohort are unclear and additional studies are warranted to investigate the different reasons why RA patients are started and kept on steroids and at what rate.

In conclusion, steroid use is prevalent in patients starting b/tsDMARDs and remains frequent over the first year of treatment. This disproportionally affects patients with severe RA where multiple previous treatments were not successful. More research and clinical trials are required to understand the best and safest course of steroid treatment to maximize therapeutic effects.

## Supplementary Material

rkaf130_Supplementary_Data

## Data Availability

The data underlying this article cannot be shared publicly for the privacy of individuals who participated in the study. The data will be shared upon reasonable request to the corresponding author.
