# Supplementary material for: Patterns of steroid use in patients with established rheumatoid arthritis commencing treatment with biologic or targeted synthetic DMARDs
Source: Rheumatol Adv Pract. 2025 Dec 22;10(1):rkaf130. doi: 10.1093/rap/rkaf130 (PMC12758120; doi:10.1093/rap/rkaf130)
Supplement: rkaf130_Supplementary_Data [file rkaf130_supplementary_data.zip › 25-049 Supplementary Material.docx]

Supplementary material

|  | **Group** | **N** | **Overall** | **TNFi** | **non-TNFi** |
| --- | --- | --- | --- | --- | --- |
| Etanercept | TNFi | 630 | 34.1% | 49.9% | - |
| Adalimumab | TNFi | 368 | 19.9% | 29.1% | - |
| Certolizumab | TNFi | 177 | 9.6% | 14% | - |
| Golimumab | TNFi | 55 | 3% | 4.4% | - |
| Infliximab | TNFi | 33 | 1.8% | 2.6% | - |
| Rituximab | non-TNFi | 328 | 17.8% | - | 56.3% |
| Tocilizumab | non-TNFi | 172 | 9.3% | - | 29.5% |
| Abatacept | non-TNFi | 74 | 4% | - | 12.7% |
| Baricitinib | non-TNFi | 7 | 0.4% | - | 1.2% |
| Tofacitinib | non-TNFi | 2 | 0.1% | - | 0.3% |

Supplementary Table S1: Distribution of b/tsDMARDs across the cohort (TNFi: tumour necrosis factor inhibitors; non-TNFi: later stage non-TNFi agents).

|  | **Full Cohort (N = 625)** | **< 2018 (N = 549)** | **≥ 2018 (N = 76)** | **p** |
| --- | --- | --- | --- | --- |
| Oral | 315 (50.4%) | 279 (50.8%) | 36 (47.4%) | 0.1 |
| Parenteral | 287 (45.9%) | 251 (45.7%) | 36 (47.4%) |  |
| Both | 10 (1.6%) | 10 (1.8%) | 0 (0%) |  |
| Unspecified | 13 (2.1%) | 9 (1.6%) | 4 (5.3%) |  |

Supplementary Table S2: Modes of pre-baseline steroid treatment, overall and stratified by year of treatment start.

|  | **3M (N = 521)** | | **6M (N = 527)** | | **12M (N = 560)** | |
| --- | --- | --- | --- | --- | --- | --- |
| Oral | 237 | (45.5%) | 228 | (43.3%) | 199 | (35.5%) |
| Injected | 165 | (31.7%) | 185 | (35.1%) | 217 | (38.8%) |
| Both | 36 | (6.9%) | 35 | (6.6%) | 62 | (11.1%) |
| Unspecified | 83 | (15.9%) | 79 | (15%) | 82 | (14.6%) |

Supplementary Table S3: Modes of steroid treatment over the course of the follow-up period.

|  | **β (± SE)** | **OR [95% CI]** | **p** |
| --- | --- | --- | --- |
| **Intercept** | **0.225 (±0.013)** | **1.25 [1.22 1.28]** | **< 0.001** |
| FU_2_ | 0.022 (±0.014) | 1.02 [0.99 1.05] | 0.1 |
| **FU_3_** | **0.054 (±0.014)** | **1.06 [1.03 1.08]** | **< 0.001** |
| **nTNFi** | **0.216 (±0.023)** | **1.24 [1.19 1.3]** | **< 0.001** |
| nTNFi * FU_2_ | 0.046 (**±**0.025) | 0.96 [0.91 1] | 0.06 |
| **nTNFi * FU_3_** | **0.060 (±0.025)** | **0.94 [0.9 0.99]** | **0.02** |

*Supplementary Table S4: Unadjusted mixed linear effect model of steroid use based on follow-up, and type of b/tsDMARD (β: the parameter estimate and associated standard error [SE]; OR: the odds ratio and associated confidence interval [95% CI]; p: p-value from a two-tailed t-test; bold type face indicates significance [p < 0.05]; FU_2,3_: the second [6 months] and third [12 months] follow-up, with the first [3 months] serving as reference category; nTNFi: whether the patient received non-TNFi treatment [Yes/No]).*

|  | **β (± SE)** | **OR [95% CI]** | **p** |
| --- | --- | --- | --- |
| Started from 2018 | -0.055 (±0.029) | 0.95 [0.89 1.00] | 0.06 |
| **First b/tsDMARD** | **-0.067 (±0.024)** | **0.94 [0.89 0.98]** | **0.006** |
| **Pre-baseline steroids** | **0.341 (±0.016)** | **1.41 [1.36 1.45]** | **< 0.001** |
| RF positive | -0.038 (±0.02) | 0.96 [0.93 1.00] | 0.06 |
| conc. cDMARD | -0.049 (±0.03) | 0.95 [0.90 1.01] | 0.1 |
| Choice of conc. cDMARD |  |  |  |
| MTX | -0.057 (±0.032) | 0.94 [0.89 1.01] | 0.1 |
| HCQ or SSZ | -0.026 (±0.037) | 0.97 [0.91 1.05] | 0.5 |
| MTX + (HCQ or SSZ) | -0.05 (±0.032) | [0.89 1.01] | 0.1 |

*Supplementary Table S5: Baseline adjustment factors for the linear mixed effect model of steroid use across the follow-up (β: the parameter estimate and associated standard error [SE]; OR: the odds ratio and associated confidence interval [95% CI]; p: p-value from a two-tailed t-test; bold type face indicates significance at p < 0.05; Started from 2018: whether the patient started treatment before [No] or after the 2018 guidance update [Yes]; First b/tsDMARD: whether this was the patient’s first course of b/tsDMARDs [Yes/No]; Pre-baseline steroids: whether the patient had received pre-baseline steroids [Yes/No]; RF positive: whether the patient was rheumatoid factor positive; conc. cDMARD: whether the patient received concomitant conventional DMARD treatment at baseline; MTX: methotrexate; HCQ: hydroxychloroquine; SSZ: sulfasalazine).*

|  | **3M (N = 62)** | | **6M (N = 71)** | | **12M (N = 73)** | |
| --- | --- | --- | --- | --- | --- | --- |
| Oral | 37 | (59.7%) | 36 | (50.7%) | 30 | (41.1%) |
| Injected | 11 | (17.7%) | 17 | (23.9%) | 21 | (28.8%) |
| Both | 0 | (0%) | 1 | (1.4%) | 4 | (5.5%) |
| Unspecified | 14 | (22.6%) | 17 | (23.9%) | 18 | (24.7%) |

Supplementary Table S6: Modes of steroid treatment for patients who received steroids despite being in DAS28_CRP_ remission.
